# A Smarter Pathway for Delivering Cue Exposure Therapy? The Design and Development of a Smartphone App Targeting Alcohol Use Disorder

**DOI:** 10.2196/mhealth.6500

**Published:** 2017-01-30

**Authors:** Angelina Isabella Mellentin, Elsebeth Stenager, Bent Nielsen, Anette Søgaard Nielsen, Fei Yu

**Affiliations:** ^1^ Unit of Clinical Alcohol Research, Unit of Psychiatric Research Department of Clinical Research University of Southern Denmark Odense C Denmark; ^2^ Unit of Psychiatric Research Institute of Regional Health Services Research University of Southern Denmark Aabenraa Denmark; ^3^ Technology Entrepreneurship and Innovation section Mads Clausen Institute University of Southern Denmark Soenderborg Denmark

**Keywords:** alcohol use disorder, exposure therapy, mobile application, smartphone

## Abstract

**Background:**

Although the number of alcohol-related treatments in app stores is proliferating, none of them are based on a psychological framework and supported by empirical evidence. Cue exposure treatment (CET) with urge-specific coping skills (USCS) is often used in Danish treatment settings. It is an evidence-based psychological approach that focuses on promoting “confrontation with alcohol cues” as a means of reducing urges and the likelihood of relapse.

**Objective:**

The objective of this study was to describe the design and development of a CET-based smartphone app; an innovative delivery pathway for treating alcohol use disorder (AUD).

**Methods:**

The treatment is based on Monty and coworkers’ manual for CET with USCS (2002). It was created by a multidisciplinary team of psychiatrists, psychologists, programmers, and graphic designers as well as patients with AUD. A database was developed for the purpose of registering and monitoring training activities. A final version of the CET app and database was developed after several user tests.

**Results:**

The final version of the CET app includes an introduction, 4 sessions featuring USCS, 8 alcohol exposure videos promoting the use of one of the USCS, and a results component providing an overview of training activities and potential progress. Real-time urges are measured before, during, and after exposure to alcohol cues and are registered in the app together with other training activity variables. Data packages are continuously sent in encrypted form to an external database and will be merged with other data (in an internal database) in the future.

**Conclusions:**

The CET smartphone app is currently being tested at a large-scale, randomized controlled trial with the aim of clarifying whether it can be classified as an evidence-based treatment solution. The app has the potential to augment the reach of psychological treatment for AUD.

## Introduction

Alcohol use disorder (AUD) contributes to a substantial number of contacts with the treatment system [1,2], given that relapse is the most likely outcome of treatment [3-5]. Apart from being a source of suffering for affected individuals and their relatives, AUD places a significant burden on the health care system [1,2,6]. This burden is particularly prominent in the Nordic counties, Denmark being among those with the most liberal alcohol culture, leading to pervasive exposure to alcohol and associated situations. Such pervasive exposure may consequently lead to more individuals developing AUD and induce urges that can increase rates of relapse after the treatment has ended [7-9].

Within the Danish treatment system, individuals with AUD are most commonly treated with motivational interviewing, cognitive behavior therapy and family therapy, classified as evidence-based treatments [10,11]. In several Danish treatment institutions, additional cue exposure treatment (CET) is often used to reduce urges and prevent relapse in order to prepare AUD individuals to navigate in the Danish society. During conventional CET, patients are exposed to alcohol or related stimuli in vivo while their habitual drink response is hindered, so that conditioned automatic responses can be extinguished [12-14]. CET is often combined with the use of urge-specific coping skills (USCS), as there is evidence to suggest that this method provides better treatment outcomes [15-17].

When addressing the need for AUD treatment (such as CET), it is evident that the duration of the treatment is decreasing and that it is increasingly being used in group—rather than individual sessions were found appropriate and reasonable [11]. However, more individuals could potentially benefit from individual—as well as continued treatment [18,19]. There are also many individuals with AUD who never enter the treatment system [19-21], which may, in the future, cause severe collateral damage and exacerbate the burden on the health care system [1,6,9,18,20]. The implementation of e-health interventions through devices such as computers, tablets, and smartphones represents a new pathway for treatment delivery, one which overcomes some of these issues and assures accessibility to as many patients as possible nationwide [22-24]. Yet, very few of the currently available eHealth interventions are based on a theoretical framework and experimental evidence [22,25-27]. Less is known about evidence-based mobile devices, such as smartphone apps [26,28,29]. Dedert et al (2015) recently conducted a systematic review on eHealth interventions targeting AUD, revealing a huge gap in experimental evidence; they identified only a single randomized controlled study that investigated a mobile device [26,30].

Mobile eHealth interventions have the potential to play a crucial role in the future provision of continuing care and relapse prevention helping to lower the socioeconomic burden on the health care system by decreasing the number of contacts it gets, as well as augmenting the reach of relevant treatment. However, there is a need for more transparency regarding the underlying psychological framework of mobile eHealth interventions, their design, and development, as well as the provision of evidence to gain more knowledge about their effectiveness.

In order to add to the evidence base for mobile eHealth interventions, a CET smartphone app that mimics CET with USCS was designed and developed and is currently being tested in a large-scale, randomized controlled trial (ClinicalTrials.gov NCT02298751) [31,32].

The objective of this paper was to describe the design and development of a manual-based smartphone app that mimics CET with USCS, which is currently being delivered in Danish inpatient and outpatient clinics. The CET app has the potential to contribute to the reach of evidence-based psychological treatment for AUD.

## Methods

CET features in treatment programs being used in Danish alcohol clinics in both inpatient and outpatient treatment settings. CET is most commonly used in combination with various urge-specific coping-strategies (USCS), due to the promising outcomes shown [15-17]. When developing the CET app, we applied Monti and colleagues’ (2002) treatment manual for CET with USCS, which emphasizes the importance of “confrontation with alcohol” in diminishing cue reactivity. According to the treatment manual, patients were introduced to a USCS during each CET session and were thereafter required to train the learned strategy while being exposed to alcohol in vivo [33]. Due to the highly structural properties of this treatment and our clinical experience with using it, we were able to convert it into a smartphone app.

The initial plan for the structure and content of the app was developed by a group of psychiatrists and a psychologist relying on the aforementioned manual. When converting the manual, designing the app as simply, intuitively, and feasibly as possible was of utmost importance, given that the target population may have very different cognitive profiles [34-36]. Although patients with severe cognitive impairments are not candidates for this type of treatment, some of our patients might have had mild to moderate cognitive impairments after years of suffering from AUD. In accordance with the plan, programmers and graphic designers developed a preliminary version of the CET app. After several modifications and user tests with the involved psychiatrists, psychologist, and programmers, a more detailed structure of the program was confirmed. Hereafter, the app was presented to 2 patient focus groups (2×n=5) who gave feedback. All patients found the app to be simple, intuitive, and feasible. Suggestions for improvements centered mainly on the used terminology. A final version of the CET app was developed based on the patients’ feedback and is currently being tested in the previously mentioned Cue Exposure study [31], which is part of the RESCueH studies [32].

Along with the smartphone app, an online database was designed and developed in the system which can monitor patients’ data in real-time.

The open-source Linux-based operating system Android was selected as the platform for developing the smartphone app. A customized version of Java in Eclipse (Oracle Corporation) was used as the main programming language. An online server is registered for the database and monitoring of the treatment process remotely.

## Results

### The Structure and Content

**Figure 1:**
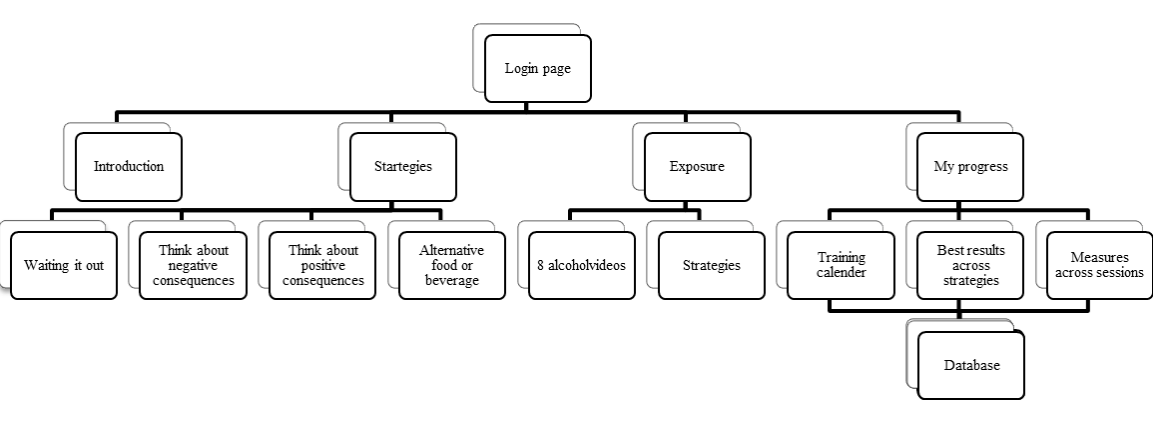
Structure of the cue exposure treatment (CET) app.

Figure 1 illustrates the structure of the app and its main content comprising the following components: introduction, 4 sessions with USCS, 8 alcohol exposure videos featuring guidance for applying one of the USCS, and a results component providing an overview of training activities and potential progress.

The information in the app is presented in text format and read out loud simultaneously.

The software requires patients to train on a regular basis and sends a text reminder for this. As little is known about the effectiveness of intensive CET [15-17,37-42], patients are allowed to train only once a day for a maximum of 4 weeks in order to prevent overexposure. The specific components of the app are outlined in the following sections.

#### Delivery and Access to the App

A CET therapist provides patients with both oral and written information about the app prior to the commencement of treatment. Patients can download the app directly onto their smartphones if they already have one. Otherwise, they can borrow a smartphone from the alcohol treatment clinic.

**Figure 2:**
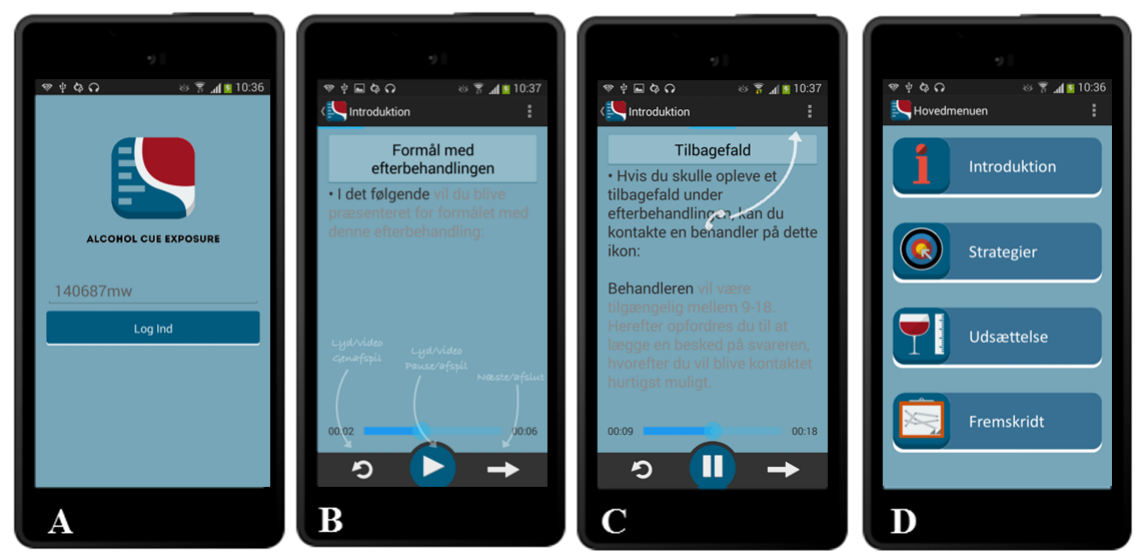
Log-in page (part A), introduction (part B and C), and main menu (part D).

As can be seen on the *Log-in* page (part A), patients are provided with a user ID that is easy to remember and that assures anonymity, permitting them to login to the CET treatment program (Figure 2, part A). We predefine all user IDs in the form of a combination of ciphers and letters, for example, 001001aa, 002002bb. Considering that some patients may have mild to moderate cognitive impairments (eg, impaired memory), we designed the login procedure to incorporate a user ID that doesn’t require a password, thus simplifying the login process. User IDs do not resemble one another so as to avoid double usage by patients logging in on another patient’s user ID. In addition, phone stickers displaying contact numbers for technical and treatment support are given to patients in case they forget their user ID, or if other technical or therapeutic issues arise during treatment.

#### Introduction to CET

The *Introduction to CET* component plays automatically the first time the app is activated and a patient is logging in. The purpose of the Introduction is to inform patients about the purpose and content of the CET with USCS, technical functions, as well as key safety functions (Figure 2, parts B and C). The Introduction to CET emphasizes that it may indeed be difficult to avoid being exposed to alcohol in Denmark, and that the purpose of using the app is to learn how to cope with cue-induced alcohol urges and associated situations in order to prevent relapses when outside the treatment setting. Hence, the treatment consists of teaching coping strategies to reduce urges, and, by exposing patients to alcohol in vivo, it trains them to tolerate urges by using the USCS.

The technical functions such as *audio/video replay*, *audio/video pause/start*, and *continue to next page*, are illustrated through arrows explaining how they work (Figure 2, part B). The safety function’s main component consists of a call icon (at the upper right-hand side of the screen) connecting to a CET therapist (Figure 2, part C), which becomes available whenever the app is activated. The call icon provides the same contact numbers as displayed on the phone stickers, hereby assuring that patients can still get in touch with a therapist even if they lose their sticker or for any other reason are not able to use the call icon. The therapist is available Monday to Friday from 9:00 am to 18:00 pm, and should be consulted in the event of experiencing uncontrollable urges. For practical and safety reasons, the app is closed for use on weekdays from 18:00 pm to 9:00 am and during the weekend, that is, when the therapist is out of reach.

If patients wish to replay the Introduction, they can click on the icon illustrated in the *Main menu* (see Figure 2, part D), which is also where they are directed to when logging-in in the future. Patients are ready to proceed to the USCS sessions after the Introduction has played.

#### Sessions With USCS

**Figure 3:**
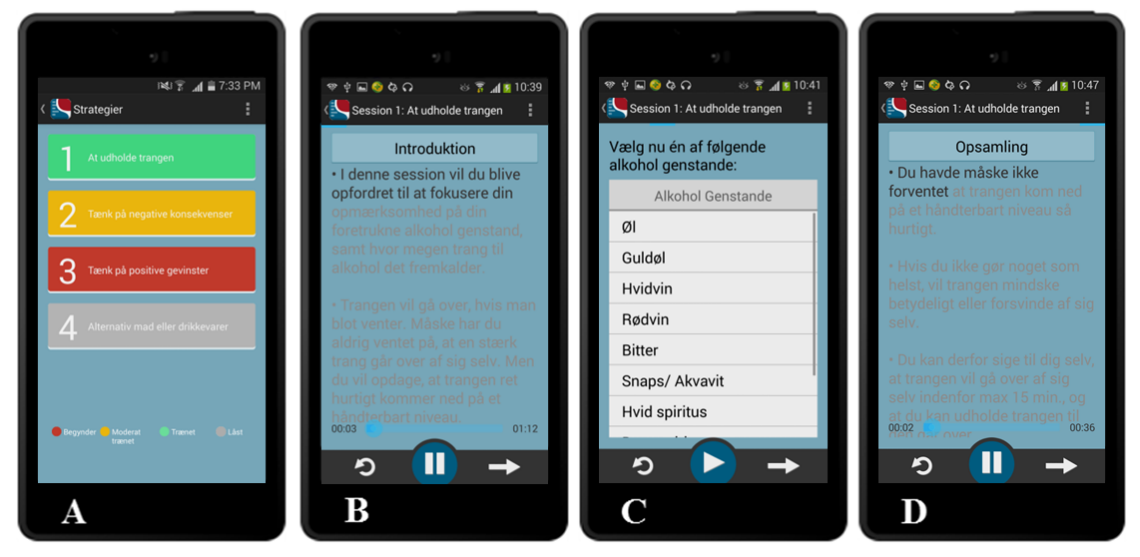
Strategies (part A); session 1: waiting it out, introduction to the USCS (part B), selection of exposure video (part C), and session 1: waiting it out, summary of the USCS (part D). USCS: urge-specific coping skills.

As shown in Figure 3 (part A), the *Strategy icon* comprises 4 sessions, each promoting the use of 1 of the 4 USCS recommended in the manual. Each session starts with an introduction to the USCS and an explanation for how to apply it during alcohol exposure (Figure 3, part B). Patients are then required to select an exposure video (Figure 3, part C). At the end of the exposure video, a summary of the USCS training and how to use the USCS in the future is provided (Figure 3, part D).

T he recommended strategies are as follows:

##### Waiting It Out

This is used as a cognitive strategy, whereby patients are explained what to expect when waiting out an urge. It is elucidated that they perhaps haven’t ever waited for a strong urge to pass naturally, and that urges actually reduce quite quickly to a manageable level. The urge passes if one waits. It can be expected to peak within 4 min, start to decline after approximately 8 min, and fall completely within 10-15 min. Moreover, the urge declines faster each time exposure occurs without resulting in alcohol consumption. This approach stands in contrast to the exclusively behavioral version of CET in which no information is provided about what to expect.

##### Thinking About the Negative Consequences of Drinking

Patients are encouraged to imagine the most negative future consequences associated with resuming alcohol abuse. In order to systemize and register the negative consequences in the database, patients are required to select between 1 and 3 consequence categories from a list comprising physical health, mental health, family and friends, work and education, economy, offences, and loss of control. To the best of our knowledge, these categories incorporate the vast majority of negative consequences that individuals with AUD may experience [43-46]. After selecting the consequence categories, patients are guided in rehearsing the USCS. It is emphasized that the USCS has proven to be particularly effective when one is experiencing predominantly positive emotions and feelings, leading to permissive thoughts about alcohol consumption. It may be useful in this situation to think carefully about the future negative consequences associated with reverting to old bad habits.

##### Thinking About the Positive Consequences of Sobriety

Patients are encouraged to imagine the positive future consequences associated with restraining from alcohol abuse. In accordance with session 2, patients are required to select between 1 and 3 consequence categories from a list comprising the same domains, and are also guided in rehearsing the USCS. In contrast to the prior USCS, it is explained that this strategy is effective when negative emotions and feelings prevail, and when one has the urge to drink in order to distance oneself. In such a situation, it may be useful to think of the positive consequences that lie ahead if the urge is resisted.

##### Alternative Food and Beverage

In this last session, patients are encouraged to consume alternative food and drink during exposure in order to reduce urges. It is explained that people have a tendency to prefer the food and drink that is most readily available in risky situations, and that it is a good idea to distinguish between 2 types of risk situations: (1) Alone or alone at home after work, watching TV, bored, and (2) Social events: after work, with friends, or celebration. Patients are encouraged to choose healthy alternatives that will form the basis for new habits.

In the *Summary* of every session, it is recommended that patients use the associated coping strategy when confronted with alcohol and risk situations in real life; however, in line with the safety functions featured in the *Introduction to CET* component, they are also advised against actively seeking out risk situations.

To ensure that each strategy is trained at least once, it is not possible for patients to proceed to the next session until they have completed the previous session. While the strategies are being trained, the *Session* icons change their colors from red to yellow and then from yellow to green, to indicate not trained, moderately trained, and trained.

The *Exposure icon* remains locked until all strategies have been trained.

#### Exposure

**Figure 4:**
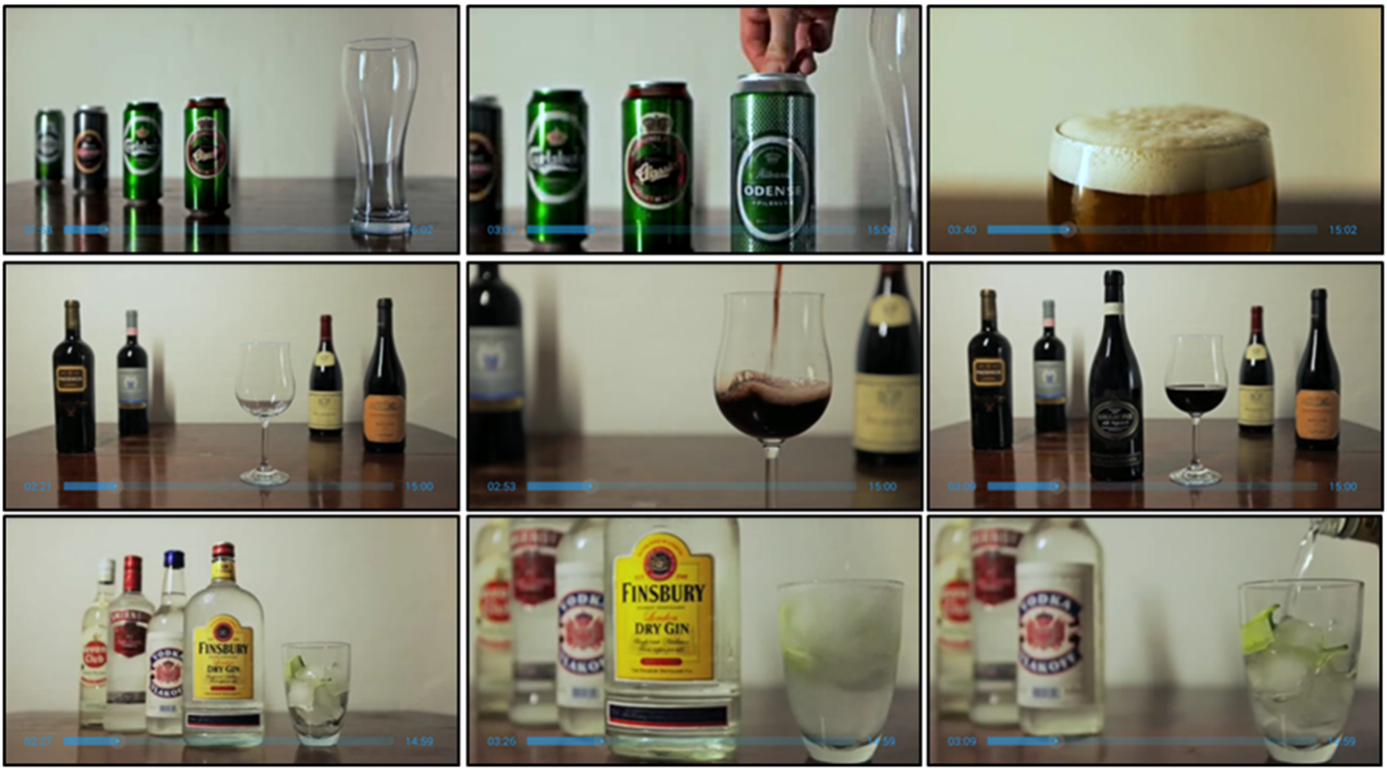
Examples of the alcohol exposure videos.

As illustrated in Figure 4, exposure to alcohol is simulated by alcohol videos.

The app contains 8 different alcohol videos comprising the following categories: ordinary beer, strong beer, alchopops, red wine, white wine, brown spirits (eg, whiskey and cognac), white spirits (eg, vodka and rum), and hard liquor. One of these should be selected. Patients can select for their preferred beverage to feature in the exposure material or vary the beverage used from one session to the next. The alcohol video is selected from the list presented in Figure 3, part C. The alcohol exposure videos imitate sessions with a therapist, and the alcohol in the videos becomes increasingly more appetizing during the exposure session so as to induce cue-controlled urges. A variety of the most common brands in Denmark are presented, as individuals with AUD have different alcohol preferences within the alcohol categories. The duration of each exposure video is 15 min. After 4 min of exposure, patients are guided in how to use the learned USCS in order to reduce the cue-induced urge. When the urge decreases to a manageable level (urge level≤2), the exposure can end, and patients can then proceed to the summary session. However, a minimum of 8 min of exposure is required.

It is possible for patients to go directly to the exposure videos after they have been trained for all the USCS, as there is no need to repeat the abovementioned information to them every time they watch a video. When patients click on the *Exposure icon*, they must register which USCS they want to train. They can then proceed to the exposure session.

#### Real-Time Measures of Cue-Induced Urges

**Figure 5:**
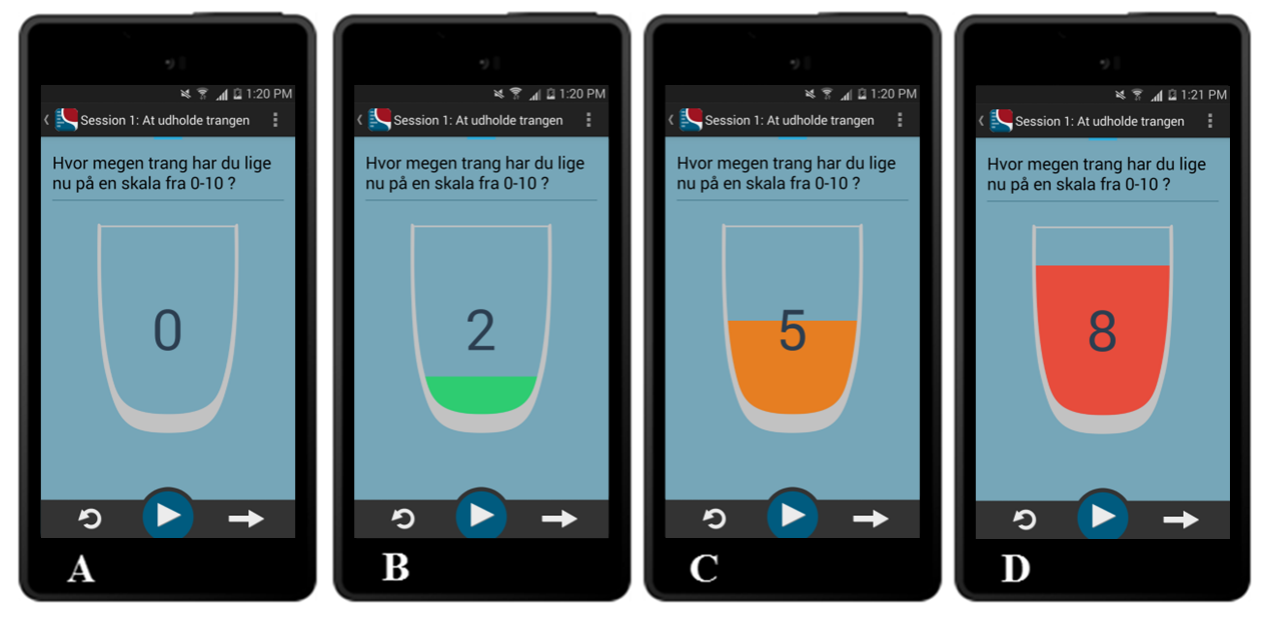
Real-time measures of cue-induced cravings.

Real-time cue-induced urges are measured at 3 time points: (1) at the baseline (before exposure), (2) when the urge is expected to peak (during exposure/4 min), and (3) at the endpoint (after exposure). Urges are measured on an 11-point Likert scale, ranging from 0 (no urges) to 10 (severe urges). As can be seen in Figure 5, we chose to use an unconventional glass-Likert scale to animate the ratings more. The liquid in the glass changes color in accordance with urge ratings. The cut-offs are 0-2 for Green, 3-6 for Yellow, and 7-10 for Red.

Based on these measures, 3-point graphic illustrations of urge development during exposure can be produced. Proxy measures of the intensity of the urge induced by the selected exposure video and the effectiveness of the selected USCS in reducing the urge can also be calculated. The first measure is calculated by subtracting the baseline measure from the peak measure. The effectiveness of the USCS is calculated by subtracting the endpoint measure from the peak measure. These algorithms together with other training activity variables are used to calculate the results in *My progress*.

##### My Progress

As illustrated in Figure 1: Main menu, the final icon is named *My progress*. My progress displays several measures and graphs allowing patients to keep track of their training activities and potential advances in controlling cue reactivity.

**Figure 6:**
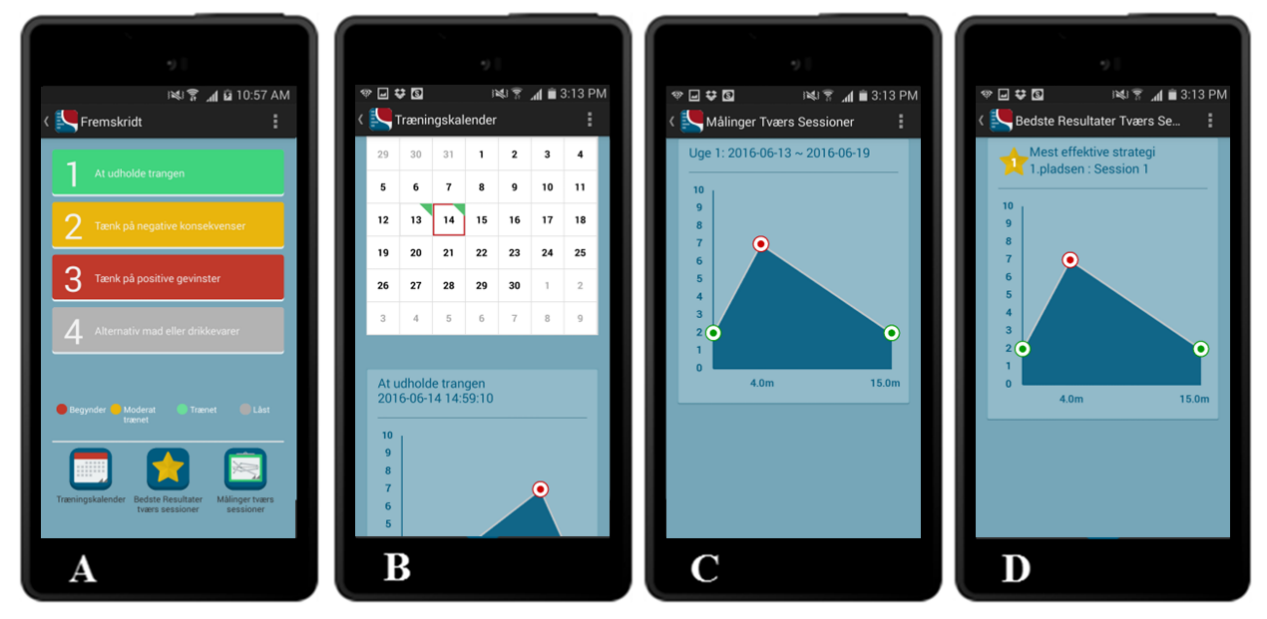
My progress (part A), calendar (part B), best results across strategies (part C), and measures across strategies (part D).

A s shown in Figure 6, the menu is similar to that in Figure 2: Sessions. Patients can click on 1 of the 4 USCS and view real-time measures and graphs related to the chosen strategy. There are 3 icons below the bars: (1) A calendar, displaying information about exposure training; (2) best results across strategies, providing information on which 3 USCS have had the best effect till date; and (3) measures across strategies, allowing access to recommendations for potential improvements regardless of the chosen strategy.

Data from these measures are recorded in the database in order to register and monitor training activities. The data would be used to measure the extent to which each USCS is applied by patients, as well as the effectiveness of each USCS and the CET intervention in general.

##### Monitoring Use and Urges

Along with the smartphone app, an online database was designed and developed in the system which can monitor patients’ data in real-time. After the patients have used a USCS, the app saves the data package locally on their phones and directs them automatically to the online database (as long as there is Internet connection). The data package includes *user ID*, *time of using the app*, *applied strategy*, and the *real-time urge data*. As already mentioned, the user IDs that we use to identify patients do not contain any personal information and are encrypted when being transferred through the Web domain to the database. The external webhosting provider, DanDomain A/S, DK, is responsible for the server and the database, and has signed an agreement with the hosts of the project to ensure that rules regarding safety and ethics are met. The database cannot be accessed by any members of the research group before all data have been collected; only contracted data managers have access to the database at this time. Any access and changes made to the database is recorded and documented.

The user IDs will be used to merge data from the database with data from other sources (in an internal database) suitable for personal identifiers.

## Discussion

### Principal Findings

This paper describes the design and development of a smartphone app that mimics the CET treatment delivered to AUD patients in Danish inpatient and outpatient clinics.

Although CET along with USCS is widely used in Denmark, studies providing evidence for the effectiveness of this treatment are yet to be conducted. If we draw on the evidence from international studies, CET has, in its conventional delivery form, demonstrated superior performance to meditation and relaxation techniques [15-17,38], and equivalent or even superior performance to cognitive behavior therapy [37,39,41,42]. Some of the best results for the effectiveness of applying CET with USCS have been reported by Monti and colleagues [15-17], and are based on the same manual that is used in most Danish treatment settings, and which this study is built on [33]. CET with USCS has been shown to work in both individual and group sessions [15,17,42].

The critical question, then, is whether this evidence-based AUD intervention demonstrates an effect when converted into a smartphone app. To answer this question, we based the present app on a behavioristic psychological framework and embedded the examination of it in a large-scale, randomized controlled trial. About 300 AUD individuals were randomized into 1 of the following 3 aftercare treatment groups: (1) CET as a mobile phone app (n=100); (2) CET as a group therapy (n=100); and (3) treatment as usual (n=100). The 2 experimental CET conditions were based on the same manual, and the treatment as usual consisted of a single follow-up session to observe how patients were doing and whether further treatment was needed. The real-time urge measures were applied in both experimental CET conditions, and a number of effect measures were conducted for all enrolled participants [31,35].

The experimental design allows for comparison between the experimental groups and the nonactive controls, which adds to the general knowledge base pertaining to the effectiveness of CET targeting AUD. Of more importance is the fact that the study design allows for a comparison between the 2 experimental conditions on USCS, real-time urges and effect measures, which clarifies whether it is beneficial for patients to progress from CET group sessions to using a CET smartphone app.

It is hypothesized that the experimental groups will achieve better outcomes compared with controls on primary and secondary effect measures, including alcohol consumption, urges or cravings and coping skills. It is a more of an explorative research question that answers whether similar or improved results for one of the CET conditions will be found. Thus, the study context will either validate or falsify the app as an evidenced-based treatment form.

Obviously, the app might have a number of disadvantages compared with CET group sessions, which may hinder its effectiveness as a pathway for treatment delivery. First, the alcohol exposure videos aim to target possibly all the individuals in the study population, hereby including several alcohol brands at the expense of the individually tailored exposure. Second, patients cannot smell the alcohol shown in the videos, and we know that smell is the only sense that is linked directly to the frontolimbic reward system [47-49]. Third, the time point for using the USCS during the exposure is based on an average for when the urges are expected to peak. Although this may be the best proxy measure, an average is an abstract value and the peak may have a broad range, hence, not capturing the real peak in many cases. Indeed, variations in the average peak have been reported across studies [14-17]. Nevertheless, both the real peak measure and the average peak measure have been reported in our CET group comparisons. This will give an estimation of the validity of the peak measure within the study population. Finally, although the app was designed to be as simple, intuitive, and feasible as possible (also for patients with minor or moderate cognitive impairments due to drinking) and a contact number for a CET therapist is provided, treatment may be affected if patients have no personal interaction with the therapist [27,50]. However, CET in app form also has several advantages. First, the CET app may facilitate extinction learning as it enables the patient to train in a variety of situations in real life. Compared with the CET treatment currently delivered in Danish alcohol clinics, this approach may increase the likelihood that extinction learning will generalize to various other contexts outside the usual treatment setting [51]. Second, the CET app is independent of time and place, and patients do not need to show up at specific times for treatment, but can instead train whenever and wherever they find it convenient. Thus, patients who have busy work schedules and family lives, live in rural areas, or have other challenges that impede them showing up regularly at the clinic may find this type of treatment more suitable. Third, apart from providing a forum for meeting the needs of AUD patients in a modern society, the application of smartphone app treatments in clinics may, also, decrease the amount of requests made for therapist-based treatment. This will, indeed, lower the burden on the health care systems’ budget. Finally, in the longer term, when evidence-based apps become more available, more patients could potentially benefit from individual- as well as continued treatment [18,19]. Moreover, AUD individuals who never enter the treatment system [19-21] could also benefit from these app services.

Although there exists a gap in knowledge about the effectiveness of evidence-based psychological treatment delivered through mobile devices [26], alcohol-related apps are becoming increasingly more available in app stores [52-54]. Worryingly, the majority of these apps are developed with the purpose of encouraging and facilitating drinking. A review of 384 apps found that only 11.5% (44/384) promoted reducing alcohol consumption to at least a moderate level of; either through providing information about detrimental effects, or through psychological interventions [54]. Although it was beyond the scope of this review to comment on the specific evidence provided by the interventions, it is doubtful that these apps are based on theory and empirical data (eg, hypnosis and motivation messages) or even guidelines. Similarly, another recent review of 662 apps found that 13.7% (91/662) targeted a moderate level of alcohol consumption. In contrast to the former review, the authors of the latter review assessed whether the promoted behavior change techniques were theory-driven and empirically validated, and found that none of them were based on theory or empirical evidence from the randomized controlled trials [52].

Despite the lack of availability of theory-driven and empirically supported apps, many new intervention initiatives targeting both subclinical and clinical AUD populations are seen in research [30,55-58]. These, as well as the present app, may contribute to the reach of more appropriate treatment in the longer term. Indeed, we are most likely witnessing a paradigm shift where delivery pathways for evidence-based treatment are progressing from individual and group sessions to (partial) mobile apps and similar delivery pathways (eg, tablets and computers) [22,24,59-61]. The delineating of these eHealth interventions is independent of time and place and could potentially contribute to reductions in problematic addictive behaviors and associated damage to a broad range of populations. However, in order to answer the question of whether mobile devices are a smarter pathway for delivering psychological treatment when targeting AUD, there is still a need for extensive research, as it is currently only in its early stages. This question will be further addressed by upcoming research in this fast growing area of study.

### Conclusions

It is our hope that the present CET app will contribute to the availability of evidence-based mental health apps targeting AUD. Future work will customize the CET app according to the findings generated by the longitudinal randomized controlled trial in which the examination of this app is embedded in.

## Abbreviations

AUD: alcohol use disorder
CET: cue exposure treatment
USCS: urge-specific coping skills
